# A Review on Montmorillonite-Based Nanoantimicrobials: State of the Art

**DOI:** 10.3390/nano13050848

**Published:** 2023-02-24

**Authors:** Syed Imdadul Hossain, Ekaterina A. Kukushkina, Margherita Izzi, Maria Chiara Sportelli, Rosaria Anna Picca, Nicoletta Ditaranto, Nicola Cioffi

**Affiliations:** 1 Chemistry Department, University of Bari Aldo Moro, Via E. Orabona 4, 70126 Bari, Italy; 2 CSGI (Center for Colloid and Surface Science) c/o, Department of Chemistry, Via Orabona 4, 70125 Bari, Italy

**Keywords:** antimicrobial, Ag, Cu, ZnO, montmorillonite, chitosan, food packaging, ion exchange

## Abstract

One of the crucial challenges of our time is to effectively use metal and metal oxide nanoparticles (NPs) as an alternative way to combat drug-resistant infections. Metal and metal oxide NPs such as Ag, Ag_2_O, Cu, Cu_2_O, CuO, and ZnO have found their way against antimicrobial resistance. However, they also suffer from several limitations ranging from toxicity issues to resistance mechanisms by complex structures of bacterial communities, so-called biofilms. In this regard, scientists are urgently looking for convenient approaches to develop heterostructure synergistic nanocomposites which could overcome toxicity issues, enhance antimicrobial activity, improve thermal and mechanical stability, and increase shelf life. These nanocomposites provide a controlled release of bioactive substances into the surrounding medium, are cost effective, reproducible, and scalable for real life applications such as food additives, nanoantimicrobial coating in food technology, food preservation, optical limiters, the bio medical field, and wastewater treatment application. Naturally abundant and non-toxic Montmorillonite (MMT) is a novel support to accommodate NPs, due to its negative surface charge and control release of NPs and ions. At the time of this review, around 250 articles have been published focusing on the incorporation of Ag-, Cu-, and ZnO-based NPs into MMT support and thus furthering their introduction into polymer matrix composites dominantly used for antimicrobial application. Therefore, it is highly relevant to report a comprehensive review of Ag-, Cu-, and ZnO-modified MMT. This review provides a comprehensive overview of MMT-based nanoantimicrobials, particularly dealing with preparation methods, materials characterization, and mechanisms of action, antimicrobial activity on different bacterial strains, real life applications, and environmental and toxicity issues.

## 1. Introduction

Antimicrobial resistance (AMR) is one of the greatest threats to public health and the economy. Resistance of microorganisms towards antibiotics, antifungals, or disinfecting agents poses a great concern in scientific communities. The World Health Organization (WHO) outlines that antibiotic resistance is one of the top 10 public health threats [1]. Bacteria develop resistance and have the ability to survive harsh environments, forming biofilms. Biofilms are communities of microbes which can be formed on living surfaces such as skin, lungs, and bladder, or non-living surfaces such as wound dressings, medical implants, and devices (artificial hearts, catheters, and stents), and food packaging. [2]. Bacterial biofilms cause infections due to their intrinsic tolerance to antimicrobial therapies. Eradicating bacterial biofilms is a challenging task, owing to the formation of dense bacterial communities with a complex architecture, which provides protection from antibiotics and antibacterial agents. One of the strategies to inhibit biofilms is the development of novel nanoantimicrobials (NAMs) [3]. In fact, over a period of decades, research attention has been driven to synthesize novel antimicrobial agents by developing fast, environmentally friendly, easily scalable, cost effective, and facile approaches [4,5,6,7,8,9,10]. Several types of metal and metal oxide NPs such as Ag, Ag_2_O, Cu, CuO, ZnO, TiO_2_, Si, SiO_2_, Au, CaO, and MgO are considered as potent antimicrobial agents [11,12,13,14,15]. Among them, Ag-, Cu-, and ZnO NPs exhibit excellent antibacterial activity and have several advantages such as improved stability and safety, and longer active periods than that of organic nanomaterials (even at low concentrations with their multi-targeted mechanism of action) [15,16,17]. However, the direct use of these NPs may lead to instability, severe reactivity, and toxicity due to their high surface-to-volume ratio, and fast and uncontrolled release properties. Moreover, metal NPs can suffer from agglomeration which eventually leads to chemical and physical changes. As a result, antibacterial activity may be reduced [18]. Therefore, it is needed to overcome those shortcomings by developing sustainable, cheap, and environmentally friendly methods to prepare novel composite nanomaterials.

In general, the antimicrobial activity of metal nanoparticles is related to their size, shape, and ion release properties. Specifically, AgNPs, CuNPs, and ZnONPs can act as antimicrobial agents because they are able to go through the cell wall, if they are small enough (<10 nm) [19]. At the same time, all of them can work as bioactive ion reservoir. It is well known that AgNPs not only act as silver ion reservoirs but also provide a high enough concentration of silver antibacterial species in their surroundings. At the same time, all of them can work as bioactive ion reservoirs. AgNPs have the ability to adsorb onto the bacterial membrane, causing “pits” in the cell wall and consequent apoptosis. Therefore, antibacterial activity remains high for a long period of time [20,21]. On the other hand, the Cu(II) oxidation state of copper is highly effective towards microbial cells, owing to interaction with nucleic acids, enzyme active sites, and components of the cell membrane. As a consequence, the microbial cells end up dying [22]. It is also proven that ZnO produces hydroxyl radicals, which lead to bacterial cell damage [23].

The European Food Safety Authority (EFSA) highlights the use of antimicrobial NPs in food packaging. In particular, food supplements which contain AgNPs must not exceed 0.05 mg/L in water and 0.05 mg/Kg Ag migration in food [24]. Moreover, other factors such as size and shape of NPs, coating, life cycle, dosing, particle agglomeration, use of biosafe polymers and solid supports, control ion release, and synthetic route, play a vital role to address cytotoxicity issues and the antimicrobial activity of NPs for real life application by designing novel hybrid synergistic materials. Using NPs instead of ions is more effective because the latter deplete rapidly, even though their initial activity is higher. Therefore, the use of ions enables an immediate and high antimicrobial activity, which can be lost in a very small lapse of time. On the contrary, NPs act as a reservoir of ions with a possible control on the leaching rate and amount, ensuring focused and long-term activity of the nanomaterials [3,25]. The use of ions rather than the metal NPs as bioactive agents is sometimes preferred, because the release and associated potential accumulation of metal NPs in the organism could be dangerous, particularly if NP size is smaller than 10 nm. In this case, Cu ions are found to be less toxic than Ag ions [26]; however, the ecotoxicity of ions and NPs released from nanocomposites needs to be researched more, considering environmental health safety issues. Thus, choosing a carrier which can effectively lower the toxicity of Ag, Cu, or ZnO NPs and improve antimicrobial performance at a low concentration of antimicrobial NPs is vital. Apart from that, supports for deposition of nanomaterials are fundamental to control NP size, and attain improved stability with long-term activity [27,28,29,30].

Ag-, Ag_2_O-, Cu-, CuO-, and ZnO-based NPs with MMT play a vital role in shaping the current research scenario, such as fighting cross-infections and infectious diseases, providing safe food technology, and treating co-existing contaminates [31,32,33,34,35,36].

The reasons for considering Ag-, Cu-, and Zn-based particles as antimicrobial nanophases is to ensure their uniform dispersion in the supporting material and to control the release of their species. Synthesis of NPs on solid support such as MMT is highly recommended to achieve formation of practically applicable supported particles. This feature would be helpful to slow and control the release of bioactive metal ions from active films or coatings, for long-lasting antibacterial activity [37]. Moreover, incorporation of colloidal NPs into the MMT matrix could lead to the development of perspective additives for real life application. MMT is an ideal substrate for preparing 2D materials for its sheet-like morphology. MMT can be exfoliated into nanosheets used in a wide range of applications as two dimensional (2D) material. Metal NP–clay–polymer composite find potential applications in food packaging and biomedical fields, due to the formation of exfoliated nanocomposite heterostructures with a random dispersion of bioactive metal NPs on the polymer matrix, limiting the diffusion of metal ions, and slowing down their release, reducing water and oxygen permeability, and modifying antimicrobial activity [38,39,40]. In particular, the bacterial cell wall contains lipoprotein, which exhibits a net negative charge under physiological conditions because of the presence of functional groups such as phosphate, carboxyl, and hydroxyl. On account of that, metal ions are adsorbed on the cell wall by electrostatic attraction, causing shrinkage of the cytoplasm membrane or detachment of the cell wall, resulting in inactivation and inhibition in cell processes [17,41]. In this scenario, strong metal retention capacity of the solid support improves antimicrobial activity, which could be achieved by tailoring weak hydroxyl groups of polymer–metal interaction, and an optimum amount of metal-loaded polymer onto MMT substrate [42]. The adsorption of bacteria (Coliphages T1 and T7 of *E. coli*) on MMT clay was investigated for the first time by Schiffenbauer M. and Stotzky G. [43]. MMT clay as a natural material has always been worthy of consideration and has attracted intensive attention as a novel support, it absorbs a variety of materials on its surface because it has the advantage of a lamellar structure, a large surface area, and exhibits a negative surface charge (−60 ± 10 mV) [44,45,46], is biologically inert and it is effortless to remove it from the human organism [47]. MMT/metal NPs composites exhibit excellent adsorption, rheological, ion exchange, heat insulators and are heat-resistant, and swelling properties [48,49]. On top of that, nanosheets of MMT act as a stabilizing surface for the deposition of NPs [50], which prevent NP agglomeration. MMT has the capability of accommodating NPs in their lattice which open new perspectives in manufacturing medical devices, the textile industry, wastewater treatment, drug delivery, and food packaging application. MMT is not only cost effective and environmentally friendly, but also has ability to be pillared with metal ions, forming so-called Ag/MMT, Cu/MMT, or Zn/MMT nanocomposites, allowing for improved antibacterial activity [51,52,53,54,55]. However, in most of the cases, before the incorporation of MMT into materials, it is necessary to modify it by physical (grinding and calcination) or chemical exfoliation processes, in order to improve its surface area, changing particle size, and modifying surface charge, to improve its compatibility with other materials. To this aim, several exfoliation approaches have been investigated. The use of quaternary ammonium surfactant such as cetyltrimethylammonium bromide, which causes MMT clay to become partially organophilic; the surface potential of MMT shifts from negative to positive, facilitating the dispersion and occlusion of NPs in the MMT interparticle pore, and providing effective mutual interaction between NPs and microorganisms on the surface of MMT clay. This is also due to higher homogeneity and improved diffusion properties [56,57,58,59,60], and the introduction of functional groups on MMT surfaces [61]. It is imperative to perform washing and refining steps to make the MMT matrix pure and suitable for the accommodation of metal and metal oxides into/onto its layer/surface. Physical purification such as grinding for size fractionation separates some impurities while chemical dissolution–physical purification is highly recommended to separate most of the impurities from the MMT [62]. The capability of MMT for adsorbing target metals is a distinct advantage for efficient mechanisms which can be further adjusted based on the characteristics of NPs. MMT play significant roles in supporting metal and metal oxide NPs, acting as ligands and protecting agents for reactive and uncontrolled conditions, providing an active surface site which contributes directly to the reactivity of NPs for perspective application such as catalysts or antimicrobial agents [63,64].

Recently, MMT-based two-dimensional (2D) composite materials, such as MMT-layered double hydroxide and MMT–graphene have been reviewed focusing on their preparation strategies and applications in pollution adsorption, medical antibacterial, film thermal conduction, and flame retardant [65]. In 2016, Giraldo et al. critically reviewed an Ag incorporation technique into MMT phyllosilicates and further outlined that functionalization of the MMT can promote the Ag^+^ adsorption [66].

There are several reviews on Zeolite-supported silver [67,68,69,70,71] but no comprehensive review on NP-impregnated MMT dealing with Ag, Cu, and ZnO are available. In this review, we have focused our attention on preparation methods, potential antimicrobial activity, real life application, and environmental and toxicity issues of MMT-supported Ag, Cu, and ZnO nanoantimicrobials (NAMs). We aim to collect a library of available studies since the past two decades on the improvement of an antimicrobial effect of Ag-, Cu-, and ZnO-incorporated MMT-based composites and understanding the mechanisms involved in growth inhibition. Furthermore, their introduction into the polymer matrix dominantly for antimicrobial application is discussed using the data from >200 publications.

## 2. Nanoantimicrobials Based on Various Synthetic Routes

Several methods such as chemical, physical, biological, electrochemical, and hybrid methods have been applied for the preparation of Ag, Cu, and ZnO NAMs. Each method has its pros and cons. Details can be seen in the following reviews, which particularly deal with the synthesis, mechanism, characterization, and potential application of Ag, Cu, and ZnO NAMs [19,69,72,73,74,75]. In the following sections, the different studies dealing with MMT-supported Ag, Cu, and ZnO NAMs have been classified and characterized based on preparation methods, namely, ion exchange and chemical reduction, hybrid, irradiation, electrochemical, plasma resonance, and radio frequency (RF) approaches. Additionally, the study of adsorption, structural investigation, and molecular modelling of MMT-supported NAMs have been briefly discussed.

### 2.1. Ag/MMT Nanocomposite by Ion Exchange and Chemical Reduction

As MMT is negatively charged, it can adsorb metal cations, which could be easily incorporated by simple ion exchange methods within the interlayer space of MMT, and this can eventually result in improved antimicrobial behavior with a more controlled release of the active species. For example, in 2009, Ohashi et al. generated a silver chelate complex of 6-benzylaminopurine (Ag^+^(6-BAP)_2_) MMT by cation exchange reaction in aqueous medium. The planar Ag^+^(6-BAP)_2_ ions were arranged parallel to the silicate layers. In this approach, most of the interlayer cations are replaced by the silver chelate complexes. The interlayer silver chelate complex is decomposed between 250 °C and 400 °C, with increasing temperature, the Ag^+^ ions are reduced to form silver particles by carbothermal reduction. The slow release of silver chelate is attributed to the electrostatic adsorption state in the deep part of the nanolayers [76]. Following a similar approach, in 2015, Sohrabnezhad and Sadeghi prepared Ag_2_CO_3_-Mobile Crystalline Material (MCM-41) and Ag_2_CO_3_/MMT, and claimed that AgNPs are more stable in Ag_2_CO_3_/MMT than in Ag_2_CO_3_/(MCM-41). However, Ag_2_CO_3_/MCM-41 exhibited higher antibacterial activity towards *E. coli* [77], because of the binding of silver ions to functional groups of proteins and enzymes, which lead to cell process inhibition [78]. Citing a few related works, [79,80] describe the effect of microparticles in drug delivery and photocatalytic activity of Ag_2_CO_3_. In 2016, Sohrabnezhad et al. addressed silver halide (AgX, X = Cl, Br, I)/(MMT) nanocomposites as antibacterial agents using the same common ion exchange method. Furthermore, the study showed that AgCI/MMT is more active than AgBr/MMT and AgI/MMT against *S. aureus*, *M. luteus*, and E coli [81]. However, Naik et al. previously explored Ag/AgCl–mesoporous silica nanocomposites against *E. coli* [82]. In the following year, the study of silver histidine-loaded Na-MMT antibacterial agent showed higher cation exchange capacity than pristine Na-MMT [83]. Considering the incorporation of nano-clay into polymer composite to modify antimicrobial functionality, Ag-modified MMT (Ag/MMT) and Ag/MMT as powder coating in epoxy/polyester resin, was reported. The Ag/MMT exhibited antibacterial activity against *E. coli* over 24 h, and it was thermally stable even after annealing at 180 °C. While in contrast, powder coatings of Ag/MMT dispersed in epoxy/polyester resin showed no antimicrobial performance towards *E. coli*, due to poor wetting of the polymer coating, which limits the diffusion of Ag ion from the coating [84]. On the other hand, a study of chitosan (CS)-based Ag/MMT not only demonstrated it to be an efficient antimicrobial agent but also offered a better understanding of the synergy between chitosan and Ag/MMT, between pH 3.6 and 6. MMT clay containing Ag reduces the water uptake of the nano-bio-composites, because Ag^+^ ions corroborate chemical and physical cross-linking between the chitosan macromolecules. As a result, the proposed material showed improved stability in water, and could overcome some limitations to chitosan commercialization [85].

**Figure 1:**
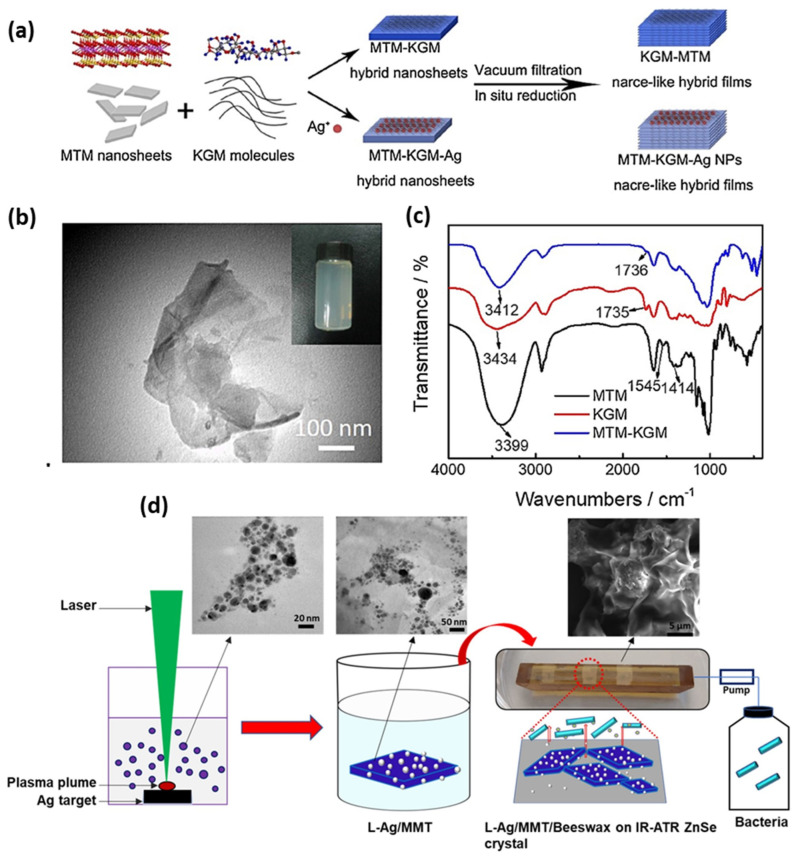
(**a**) Schematic preparation process of the composite films. (**b**) TEM images of MMT nanosheets, (**c**) FTIR spectra of MMT, KGM, and KGM–MMT composite films. Adapted from [97], with permission from Elsevier. Copyright 2018, Elsevier. (**d**) Schematic of preparation of Laser ablated Ag/MMT/Beeswax coating and IR-ATR monitoring of biofilms inhibition. Adapted from [150], distributed under the terms and conditions of the Creative Commons Attribution (CC BY) license.

Chemical reduction is one of the most common and frequently applied strategies for the preparation of NAMs in water and organic solvents. Metal NPs can be embedded on organic or inorganic substrate, with improved antimicrobial efficacy with slow and control release of NPs and ions. Basically, NPs become crystallized over substrates such as MMT layers in situ or ex situ, in the presence of added reducing agents which facilitate ions to NP transformation. Several reduction media such as chemical salts, solvents, plant extracts, and biological species are being used to control NP diameter and size distribution. In most cases, antimicrobial efficacy depends on NP size and on the nature of reducing the medium which is responsible for NPs formation on supported layers. Therefore, controlling the nature and parameters of reductants also plays a significant role in the attainment of the biocidal effect in NP/substrate nanocomposites. Conventional chemical reduction methods need special attention to make stable colloids and controlled NP sizes and shapes. Several parameters such as solution temperature, concentrations of the reducing agent, precursor and surfactant concentration ratio and mixing speed, reaction time, and the use of additional supporting materials must be optimized. Preventing NP agglomeration, and controlling their size and shape may be challenging [86]. MMT could be considered as a protective agent to prevent the NP agglomeration and to stabilize metal particles in nanocomposite [50,87]. In light of this, Praus et al. intercalated MMT with silver cations by common reduction method: formaldehyde and sodium borohydride (NaBH_4_) were subsequently used as reductants. Silver NPs with sizes ranging from 3 nm to 13 nm, and 3 nm to 100 nm were observed after reduction with borohydride and formaldehyde, respectively [88]. It is well known that antibacterial activity of Ag/MMT is higher than pristine Ag. For instance, Malachova et al. showed that the minimum inhibitory concentration (MIC) values of Ag/MMT for *E. coli* (0.87 mg L^−1^) and *Enterococcus faecium* (*E. faecium*) (1.10 mg L^−1^) are lower than the MIC value of pristine Ag for *E. coli* (0.92 mg L^−1^) and *E faecium* (1.50 mg L^−1^). The reason behind this might be due to the controlled silver ions release from MMT [51]. In the year 2010, Miyoshi et al. reported on the preparation of AgNPs (approximately 2.5 ± 0.6 nm) on MMT, in n-hexanol. Average particle size ranged from 2.5 to 6.8 nm upon changing the Ag^+^ concentration, below 0.1 M, while it reached 400 nm for [Ag^+^] = 1 M, since AgNPs coagulated to form large particles on the clay at high concentrations of Ag^+^. Nevertheless, the clay prevented further coagulation of particles and surprisingly antibacterial activity of AgNP/MMT was observed against *E. coli* even after a long time [89]. It is known that in the High-Resolution Episcopic Microscopy (HREM) setup, extremely small particles are unstable in the presence of the electron beam [90]. However, Miyoshi et al.’s group claimed that the prepared AgNPs were extremely small but stable on the MMT clay, under HREM setup. In another study, Shameli et al. generated AgNPs (5–10 nm diameter) in the lamellar space of the MMT, where NaBH_4_ and AgNO_3_ were considered as the reducing agent and Ag precursor, respectively. After that, significant antibacterial activity was found against *S. aureus*, methicillin-resistant *S. aureus* (MRSA), *E. coli* O157:H7, and *Klebsiella pneumoniae*. Additionally, it has been observed that MMT acts as both reducing and protective agent, preventing AgNP aggregation. According to the literature, it has been hypothesized that, due to their small size, AgNPs with a diameter lower than 10 nm easily penetrate inside the bacterial cell. Otherwise, they are too big and are only able to attach onto negatively charged cell walls, thus disrupting bacterial integrity, and explore a larger surface area, resulting in improving antibacterial performance [91]. However, it is arguable that, in the case of solid support such as MMT, antibacterial activity depends on the release of Ag^+^ ions from the surface of AgNPs [92,93]. In 2015, the preparation of AgNPs in the presence of CO_3_^2−^ ions was reported, Ag_2_CO_3_/MMT and Ag/MMT nanocomposite were developed in ethylene glycol, which showed antibacterial activity against *E. coli*. According to the MIC value, it is apparent that Ag_2_CO_3_/MMT exhibited higher antibacterial performance than the Ag/MMT nanocomposite because Ag_2_CO_3_ NPs were found to be well dispersed in MMT, resulting in higher silver ion release [94]. Nevertheless, there is clear argument with others finding that antimicrobial performance depends on silver content [46,95], Later, Bonga et al. synthesized AgNPs on MMT by the chemical reduction of silver nitrate with sodium citrate and measured antibacterial performance towards *S. aureus* and *E. coli*. The effect of synthetic parameters, such as higher concentration of reductant, leading to a smaller AgNP size, was investigated [96]. It is worth describing that Zhu et al. fabricated Ag-introduced nacre-like konjac glucomannan–Montmorillonite (KGM–MMT) composite films with a pearl shell micro-layer (Figure 1a) by vacuum filtration and in situ reduction method. The very first-time nacre-like structure that contains KGM was reported, where assembly of a KGM–MMT hybrid was established. Transmission electron microscopy (TEM) images as shown in Figure 1b, confirm sheet-like morphologies of MMT, indicating that the composite could be dispersed in distilled water and then aligned to a nacre-like lamellar microstructure by vacuum-filtration-induced self-assembly. On the other hand, FTIR data reveal that the hydroxyl and carbonyl group of KGM and the silicate layer of MMT has strong interaction (Figure 1c) [97]. Following the chemical reduction method, the introduction of nanomaterials within the polymeric matrices is considered as an effective approach to further improve antimicrobial activity of nanocomposite. In this sense, Shameli et al. reported the synthesis of spherical AgNPs (around 6–9 nm in diameter) on modified MMT/CS by NaBH_4_ reduction, and investigated antibacterial activity towards *S. aureus*, *E. coli*, and *P. aeruginosa* by the disc diffusion method [98]. Similarly, Roy et al. proposed that hyperbranched epoxy resins modified Ag/MMT composite shows improved antibacterial activity, mechanical, and thermal properties [99]. The investigation contributed to the preparation of multifunctional epoxy/nanocomposites [100], cellulose–hyperbranched epoxy composites for coating applications [101], Ag/TiO_2_/bentonite nanocomposites for biological application [102], and homogeneous sponge microstructures of polycaprolactone Ag/MMT/PCL where a gradual increase in Ag release properties until 4 weeks of immersion in acidified water was observed [103,104]. The bioactivity mechanism of graphene oxide–silver nanocomposites [105,106], and of Ag/cyanobacteria composites was investigated too [107]. In 2017, Li et al. also applied specific synthetic routes to the investigation of the antibacterial activity of AgNPs/dry-fabricated biofilm (DFBF), Ag/MMT, and Ag/MMT/DFBF composites against *S. aureus*, *E. coli*, and *P. aeruginosa*. The laminated structure of MMT and the network of the DFBF lowered the release of both Ag^+^ ions and AgNPs [108]. Strikingly, biosynthesis of Ag/MMT nanocomposites was presented as a green chemical route, using water plant extracts Satureja hortensis as the reducing agent, tarragon leaf extract, Ocimum basilicum; Teucrium Polium plant extract as both reducing and capping agents, and ascorbic acid as the reducing agent. The reduction in AgNO_3_ was found to be possible due to the presence of phenolic agents and proteins in the tarragon. Nonetheless, the detailed reaction mechanism is unclear. However, controlled size and uniform distribution into MMT and lecithin (MMT/LEC) were demonstrated, with antimicrobial performance against *S. aureus* and *E. coli*. According to Dairi et al., cellulose acetate/triethyl citrate (CA/TEC) nano-bio-composite film was prepared by incorporating Ag/gelatin/organically modified montmorillonite (OMMT), and thymol, where Curcuma longa (C. longa) tuber extract was used for the biosynthesis of AgNPs. The nano-bio-composite film showed antioxidant and antimicrobial activities towards bacteria and fungi; particularly, *E. coli* was the most sensitive. OMMT was considered to control silver release from the films, which outlines a long-time antimicrobial effect and the presence of thymol was useful to inhibit the growth of *E. coli* [109,110,111,112,113].

### 2.2. Cu/MMT Nanocomposite by Ion Exchange Method

Several studies have been devoted to the absorption of Cu^2+^ on montmorillonite by cation exchange. Cu^2+^ in the form of CuSO_4_, is used in large amount for antimicrobial applications; its excessive use affects environmental economic sustainability [114]. Incorporation of MMT clay could decrease the use of Cu^2+^ supplement, considering a large specific surface area of MMT efficiently adsorbs bacteria in water. In this regard, Cu/MMT nanocomposite is found to be active against *E. coli* and *S. faecalis*, *Clostridium* in broiler chicks, and jejunal contents. The improved antibacterial activity was attributed due to the attraction of Cu^2+^-exchanged MMT to bacteria cell wall by electrostatic forces [115,116,117]. Previously, the investigation of Cu/MMT nanocomposite showed strong antibacterial ability to inhibit *Aeromonas hydrophila* due to either adsorption of the bacteria from solution and immobilization on the surface of the Cu/MMT nanocomposite, or Cu^2+^ dissociation from MMT surface and exertion of their antibacterial effect on bacteria. The study suggests that the antibacterial activity of Cu/MMT nanocomposite was mainly due to the MMT surface, rather than to Cu^2+^ release [118]. Meanwhile, Tong et al. found 1024 and 2048 μg/mL MIC values for Cu/MMT against *E. coli* K88 and *S. choleraesuis*, respectively. In contrast, both bacteria still grew in the broth containing 32.768 μg/mL of MMT, which further indicated that MMT itself has no antimicrobial effect [119]. Later, the effects of Cu/MMT on intestinal microflora, digestibility, and digestive enzyme activities of Nile tilapia (*Oreochromis niloticus*) were studied [120]. In a different study, Bagchi et al. adapted an in situ reduction of copper–ammonia–complex exchanged MMT, explaining that CuNPs are both intercalated and adsorbed on MMT clay, being active against *E. coli*, *S. aureus*, *P. aeruginosa*, and *E. faecalis* with more than a 90% mortality rate after 12 h [121]. However, in case of Cu^2+^-exchanged MMT, detailed characterization including copper chemical state in the composite, copper interaction with the MMT center, and the solvation dynamics of hydroxide ions needs to be investigated for its industrial application as an antimicrobial agent. Cu/MMT composites could be used as precursors for the preparation of multicomponent hybrid synergistic NAMs.

### 2.3. Zn/MMT by Ion Exchange

Taking into account the synergy of multicomponent NAMs, Tan et al. developed Zn^2+^-Ce^3+/^MMT nanocomposite with improved antibacterial activity, which results from the increase in specific surface area and synergistic antibacterial effect. Due to the simultaneous presence of Zn^2+^ and Ce^3+^ in Zn-Ce/MMT nanocomposite, different targets on a cell membrane might be attacked and damaged at once. Ce^3+^ effectively promotes the production of more hydroxylic free radicals from Zn^2+^, which are harmful for microorganisms. The Zn-Ce/MMT nanocomposite displayed broad-spectrum antibacterial activity, possessing the MIC of 1500, 1000, 2000, and 3000 mg·L^−1^ against *E coli*, *S aureus*, *Candida albicans*, and *Mucor*, respectively [122]. In 2010, Shi et al. prepared Zn^2+^-exchanged MMT, and elucidated that Zn content is a very important factor: increasing Zn content improves the antibacterial performance of Zn/MMT nanocomposites towards *E. coli* and *S. aureus* [53].

### 2.4. Ag/MMT Nanocomposite by Hybrid Method

It is possible to modify clay surface area and cation exchange capacity by physical treatment. For instance, after calcination, bentonite adsorption capacity was found to be improved [123,124]. Hybrid methods can be considered efficient for the fabrication of NAMs with improved mechanical and thermal stability of nanocomposite, enhanced antibacterial performance, and with uniform NP distribution. Physical methods such as thermal treatment, calcination, grinding, dipping, rolling, melt combining, and stepwise assembly. could be combined with the ion exchange method. For example, Magana et al. modified physically treated (calcination and grinding) MMT with silver by the ion exchange method, to obtain Ag/MMT with a 13.3 mm mean diameter inhibition zone against *E. coli*, which was 45% larger than that of pristine MMT. After the ionic exchange, total Ag content was 3.36 and 8.37 wt% for the calcinated clay and ground sample, respectively. The MIC values of 2.5 and 1 mg/L were found for calcinated clay and ground sample, respectively. The lower MIC values obtained for the ground sample is due to the higher ionic silver content [95]. In another study, Ag^+^/MMT/suture was prepared to strengthen and improve the antibacterial activity, by the dipping and rolling approach. Ag^+^/MMT, prepared at several temperatures, showed different effects for either *E. coli* or *S. aureus*. [125]. Hybrid methods have also been considered for the fabrication of polymer-based nanocomposites. Thus, Incoronato et al. studied Ag^+^ ion-exchanged MMT, reduced by thermal treatment. The composite was embedded in agar, zein, and PCL polymer matrices, and antimicrobial activity was investigated against three strains of *Pseudomonas* spp. (foodborne bacteria) by in vitro tests. Ag/MMT NPs embedded into agar showed activity against bacteria, on account of the highest water uptake (WU) of agar hydrogel. The higher amount of absorbed water in the agar hydrogel improved macromolecular mobility for the polymeric network. As a result, small AgNPs and generated Ag^+^ were able to bind the bacterial cell wall to diffuse more rapidly toward the bacteria. However, in the case of Ag/MMT-incorporated zein and PCL polymer matrices, no substantial antimicrobial activity has been shown [126]. Similarly, Costa et al. applied ion exchange and thermal treatment to prepare Ag/MMT NPs. Following that, the activity of Ag/MMT on microbial and sensorial quality decay was investigated during the storage of packaged fruit salad (growth of mesophilic bacteria) and showed an effect of different concentrations of Ag/MMT NPs on the shelf life of fruit salad. Interestingly, a correlation was found between antimicrobial efficacy and Ag/MMT NPs concentration [127]. In the following year, the same group studied the influence of calcium–alginate coating loaded by Ag/MMT NPs on fresh-cut carrots [128]. Later on, Ibarra et al. proposed that 2-[2-(dimethylamine)]-ethoxy ethanol (DMAE) compatibilized nanocomposites exhibits uniform filler (Ag and MMT) dispersion and exfoliation [129]. Following a stepwise assembly and impregnation approach, in quaternized carboxymethyl chitosan of cetyltrimethylammonium bromide organic modified MMT silver (QCOM-Ag), composite quaternary ammonium groups were found to be more beneficial than carboxymethyl groups, to prepare silver-based nanomaterials [130]. Cetyltrimethylammonium bromide (CTAB) (Ag^+^/OMMT) filler placed into low density polyethylene could reinforce polymer NAM film and improve an effective *E. coli* bacterial reduction by as much as 70% [131]. Another study was dedicated to synthesizing quaternized chitosan (QCS)/(OMMT)/(AgNPs)(QOMA) nanocomposite by the one-step approach, and to investigate the antibacterial activity of QOMA coating on steel plates towards *E. coli*, *S. aureus*, and fungi. Moreover, physical and mechanical investigation revealed no negative effect of QOMA coating on steel plates; instead, hydroxyl groups in QOMA act as adhesive [39]. Later, migration of cetylpyridinium bromide (CPB) from the low density polyethylene/MMT/CPB composite to aqueous food simulant was studied, proposing MMT as potential surfactant carrier [132]. Golubeva et al. developed Ag/Lysozyme/MMT bio-nanocomposite, with Lysozyme (Lys) acting as an organic shell, by hybrid method. Furthermore, they proposed the potentiality of the prepared sample as antimicrobial [133]. In early 2020, Roy et al. performed in vitro and in vivo analyses of polymeric nanocomposites as antimicrobial agents for the first time. Here, polyethylene/AgMMT nanocomposite was fabricated by melt compounding route [134]. An antimicrobial test suggested biocidal activity of the polymeric nanocomposite against *E. coli*, *S. aureus*, and *A. niger*. In vitro cytocompatibility studies with human erythrocytes and histopathological studies using rat skin surgically stitched with nanocomposite films were carried out. Recently, Zhang et al. demonstrated the potentiality of copper-loaded black phosphorus nanocomposites [135].

Ag-embedded OMMT and Polyacrylonitrile (PAN)-modified Ag/OMMT nanocomposites were considered as effective antibacterial agents in a liquid phase [136,137]. Samples were found to have the potential to cultivate fungi, and the effect of PAN on graphene oxide was investigated [138], although the role and mechanism of PAN in the nanocomposites towards pathogens was not properly understood. Those studies stimulated the exploration of Ag-doped polyaniline–polyvinyl chloride nanocomposite films as either photocatalysts or antibacterial agents [139]. In Ag/MMT, Ag^+^ is bound to the inner surface [140], while the outer surface is bound to Al-OH or Si-OH by electrostatic bonding [141]. MMT-modified with soy lecithin and citronellol, OMMT modified with carboxymethyl chitosan (QC), and AgNPs, were applied as functionalized antimicrobial coatings [142,143]. Applying a solution casting method, Makwana et al. reported agar–carboxymethyl cellulose Ag/MMT/agar–CMC, where tea extract was used as the reducing agent, with Ag/MMT apparently improving the mechanical and antimicrobial properties of agar–CMC polymer films [144]. Abbasian et al. synthesized AgNPs (2–10 nm) using AgNO_3_ as a precursor, CS-grafting polymerizations of acrylic acid (g-PAA) as a stabilizer. It was prepared by reversible addition−fragmentation chain-transfer (RAFT) polymerization, and the introduction of MMT further improves the sample thermal stability. Ag/CS-g-PAA/MMTs exhibited higher activity towards *E. coli* and *C. albicans* than *S. aureus*. However, overall antimicrobial activity of Ag/CS-g-PAA was found to be higher than Ag/CS-g-PAA/MMTs. Nevertheless, the mechanism and reasons behind this apparently inconsistent performance were not fully addressed [145]. Golubeva et al. modified synthetic MMT by Ag/Lys using a hydrothermal method and explored its hemolytic activity on human erythrocytes for the first time. Further, they showed that the use of aluminum oxide on MMT eventually reduced the antibacterial activity, but improved adsorption capacities of the prepared samples [146]. It is important to highlight that hemolytic activity of silver nanomaterials towards human erythrocytes was first demonstrated by Bock and Muller [147]. Through a one-step, low-temperature solvothermal technique, Wu et al. proposed to use MMT as a support for Ag/TiO_2_ NPs; this composite was applied for visible-light bacteria photodegradation and showed good stability, recyclability, and improved antibacterial activities [148]. Meanwhile, Park et al. used an electrospinning method in order to fabricate poly-(vinyl alcohol) (PVA) hydrophilic polymer containing Ag/MMT in aqueous medium, as an efficient antibacterial agent [149]. Recently, a hybrid route (laser ablation synthesis in solution and wet chemical approach) was applied to the preparation of Ag/MMT-based antibiofilm coatings (Figure 1d). The advanced infrared attenuated total reflection (IR-ATR) spectroscopy technique allowed real-time in situ monitoring of histamine-producing *Lentilactobacillus parabuchneri* biofilms inhibition [150].

### 2.5. Cu/MMT by Hybrid Method

CuO/MMT nanohybrids open the potential to use CuO NPs as antimicrobial agents, while MMT support helps limiting toxicity issues [151]. CuO NPs immobilized into interlayers of MMT by thermal decomposition method show excellent antibacterial activity towards *E. coli*, as a result of Cu ion release while interacting with an aqueous phase. Pourabolghasem et al. demonstrated that the alkaline ion exchange method was a fast and simple route to synthesize Cu/MMT nanocomposites, which possess good antimicrobial activity against *S. aureus* and *E. coli* in broth media. A lower Cu^2+^ ion release from the nanocomposite was obtained thanks to the diffusion of Cu into the MMT, rather than the attachment of Cu NPs on MMT surface, which leads to longer antibacterial activity [152].

**Figure 2:**
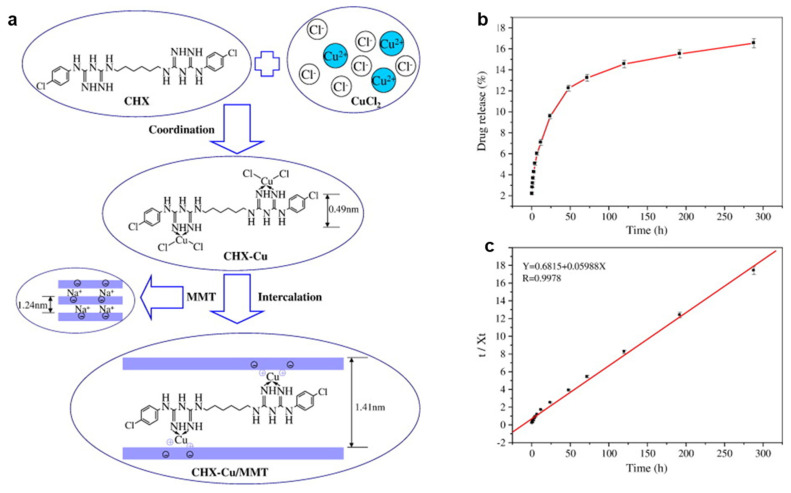
(**a**) Schematic for CHX–Cu (II) complexes formation and intercalation into the interlayer of MMT. (**b**) The release profile of CHX–Cu complexes from the CHX–Cu/MMT nanocomposite in 0.9% NaCl solution at 37 °C; (**c**) linear regression curves of release data fitting with pseudo-second kinetic mode. Adapted from [153] with permission from Elsevier (Copyright 2013, Elsevier).

The development of polymeric nanocomposites with antibacterial properties opens up new perspectives in manufacturing medical devices, the textiles industry, wastewater treatment, and food packaging applications. Thus, many researchers have been focusing their attention on a variety of peculiar synthesis routes, including the incorporation of nanomaterials into polymer matrices. In light of this, Wu et al. reported the intercalation of chlorhexidine (CHX)–Cu(II) complexes into the interlayer of MMT (Figure 2a). To maintain the neutral charge of silica layers, a large amount of sodium chloride was introduced. After intercalation at the ratio of 5 for chlorhexidine (CHX)–Cu/MMT, the basal spacing reached 0.96 nm to 1.41 nm. Therefore, the interlayer height of CHX–Cu/MMT was found to be 0.45 nm. This value was nearly equaled to the height of the phenol ring of CHX itself (0.49 nm). Furthermore, it can be seen in Figure 2b that 16% of total CHX–Cu complexes were released over 288 h from CHX–Cu/MMT nanocomposite. Further, release kinetics data (Figure 2c) indicated that the pseudo-second-order mode is consistent with the release process. On the other hand, even a 0.25 mg/mL MIC value of the CHX–Cu/MMT nanocomposites completely inhibited the bacterial growth of both *E. coli* and *S. aureus*, owing to a synergistic effect of Cu and CHX [153]. Bruna et al. synthesized Cellulose acetate (CA)/copper montmorillonite (MMT/Cu^2+^) antimicrobial nanocomposites by the solution casting method. The nanocomposite exhibited improved antibacterial performance against *E. coli*, because of the diffusion of the antimicrobial agent in the CA [38]. Later, Jaiswal et al. analyzed the benefits of the Cu^2+^/MMT/CA nanocomposite application in food microbiology [154]. In 2018, Bruna et al. prepared low density polyethylene (LDPE)/Cu^2+^/MMT nanocomposite films by ion exchange and subsequently melt mixing in an extruder at 200 °C; the study showed a reduction of 94% of *E. coli* O157:H7 colonies, whilst 4 wt.% of MMT/Cu^2+^ was loaded to the polymer [155]. Later, the same authors developed poly (lactic acid) (PLA)/Cu^2+^MMT films, which revealed a reduction of 99% of *E. coli* ATCC 25922 and *Listeria innocua* ATCC 33090 at only 5% *w*/*w* Cu^2+^/MMT loading [156]. Previously, Nouri et al. claimed CuO/MMT interlayer sites cause increased electrostatic attraction between chitosan film and bacteria. Consequently, microbial cell wall can be damaged. The introduction of MMT/CuO (3% *w*/*w*) into chitosan films reduced water vapor permeability as the number of hydroxyl groups reduced by the increase in hydrogen bonding between chitosan and CuO/MMT [157], while improved mechanical properties and antimicrobial activity towards *S. aureus* and *E. coli* were observed. However, the antibacterial mechanism of the formed sample has not yet been identified [158]. Martucci and Ruseckaite incorporated Cu^2+/^MMT into a bovine gelatin matrix via a dissolution–intercalation method to fabricate functional nanocomposite films, and postulated that blending gelatin with 5% *w*/*w* of clay improved the tensile strength of the nanocomposite films up to 280%, while the elongation at break and the water vapor permeability decreased to about 42% and 30%, respectively. The nanocomposites showed a higher antibacterial effect on *L. monocytogenes* than on *E. coli* O157:H7, due to the different nature of the *E. coli* cell wall, which influenced their response to the active films [159]. Das et al. introduced Cu/OMMT into Mesua ferrea L.-seed oil-modified epoxy resin (BPSE), to obtain clay mineral polymer nanocomposites (CPN), which showed significant efficacy towards Gram-negative bacteria (*K. pneumoniae*). Thermostability, improved mechanical properties, and enhanced antibacterial performance were also shown. OMMT in CPN acts as reinforcing nanofiller, which improves the thermal and mechanical properties of CPN. H-bonding or other polar–polar interactions between hydroxyl groups of OMMT and epoxy/carboxyl/hydroxyl groups of the BPSE lead to the formation of stable and well-dispersed CPN. The presumable clay mineral binding to the bacterial surface helps CuNPs exert their action on bacteria. The authors also observed antihemolytic behavior of the CPN to mammalian red blood cells [160]. Later, Monsif et al. studied the effect of Natural Moroccan Clays on epoxy/nanocomposites and evaluated the antimicrobial performance of the composite [100]. Bartolozzi et al. demonstrated that binary and glass laminated reinforced epoxy resin nanocomposites showed outstanding antimicrobial activity due to the presence of MMT modified with Cu/MMT [161]. Previously, an antibiotic immobilized Cu_2_O/OMMT nanohybrid was prepared using carbon dot as the reducing agent in a hyperbranched epoxy matrix. The nanocomposite was investigated as a high performance antimicrobial material towards *E. coli* [162]. Recently, Sun et al. prepared QCS/MMT/5-FCCu as a drug nanoplatform, where 5-fluorocytosine (5-FC) antibiotic was coordinated with copper ions, and QCS coating on MMT was involved. Notably, more than 120 h of 5-FCCu drug release were found. Moreover, both in vitro and in vivo studies suggested the biocompatibility properties of the nanocomposite [163]. Carboxymethylcellulose (CMC) spray-coated Cu^2+-^intercalated MMT NAMs showed excellent in vitro antibacterial efficacy against *Erwinia carotovora* in controlling potato soft rot (Solanum tuberosum), exploring the release properties of Cu^2+^ release [164].

### 2.6. Zn/MMT by Hybrid Method

In 2017, Garshasbi et al. first used an alkaline ion exchange method to prepare Zn/MMT nanocomposites, and explored that, by increasing temperature and processing time, MMT pore sizes expanded, and Zn^2+^ ion loading increased, while no impact on the antibacterial activity was noted [165]. In contrast, Zou et al. applied a hydrothermal method to prepare Zn/MMT coatings which were effective antibacterial materials with good bone cell compatibility. Additionally, in vitro MTT tests and live–dead stains of osteoblast cells revealed the improved cytocompatibility of Na-MMT and Zn/MMT coatings [166]. Recently, Wang et al. investigated the biocompatibility of Mg alloy with MMT/bovine serum albumin (BSA) coating, and its in vivo and in vitro performance [167]. Hu et al. applied sol–gel intercalation to prepare MMT/ZnO composites with improved performance in respect to bare ZnO [168,169]. Since the introduction of nanomaterials into polymer composites emerged as a promising approach to develop novel antimicrobials, Mallakpour et al. developed poly(amide-imide) (PAI)/ZnO/OMMT nanocomposites by polycondensation and subsequent solution intercalation, and investigated the effect of ZnO and OMMT on the proposed nanocomposite [170]. In 2018, Zahedi et al. developed carboxymethyl cellulose (CMC)/MMT/ZnO nanocomposite using a solution casting method, and claimed that the introduction of MMT and ZnO NPs improved the functional characteristics of CMC film, which enhanced the resistance of the CMC/MMT film to water vapor transmission owing to ZnO NP loading [171]. In order to explore the importance of NP size and their distribution effect on antibacterial performance, Chitosan-grafted MMT/polyaniline (PANI)/ZnO nanocomposite was developed [172]. Later, Ramya et al. did optical studies based on MMT/Chitosan (CS)/phenylenediamine (pPDA) composite, and proposed the composite as optical limiter [173]. Vaezi et al. studied the dispersion of MMT and ZnO NPs with different percentages in cationic starch polymer matrix. Mechanical properties of starch polymer improved upon the incorporation of 3 wt% and 0.7 wt% contents of MMT and ZnO, respectively. Further, increasing the amount of ZnO enhanced the optical properties of thermoplastic cationic starch/montmorillonite biodegradable films. Those functional characteristics are highly appreciated for food packaging application [174]. Roy et at incorporated Zn/MMT into HDPE by melt compounding route in a co-rotating twin screw micro-extruder. The nanocomposite was effective against both bacteria and fungi. Further, the effect of the Zn valence state on the antimicrobial activity was reported [175]. Similarly, Na-MMT coated with ZnO nanorods was incorporated into biodegradable antibacterial PVA polymer matrix [176]. Recently, electro-spun polyacrylonitrile (PAN)/(ZnO/MMT) nanofibrous membrane was found to be effective against bacterial strains [177]. Synergistic cobalt-doped ZnO quantum dots (QDs)/cetyltributylphosphonium bromide (CTPB)–MMT NAMs were prepared via a facile chemical precipitation method. The study suggested that Co-ZnO QDs could be absorbed on the surface and into the layers of CTPB–MMT [178]. ZnO has an intrinsic tendency to be agglomerated during practical application, resulting in reduced antibacterial activity. To overcome this issue, ZnO-loaded tetraethyl orthosilicate (TEOS)-MMT composite could improve antibacterial activity of the nano ZnO due to uniform dispersion on MMT [179].

### 2.7. Ag/MMT Nanocomposite by Irradiation Method

**Figure 3:**
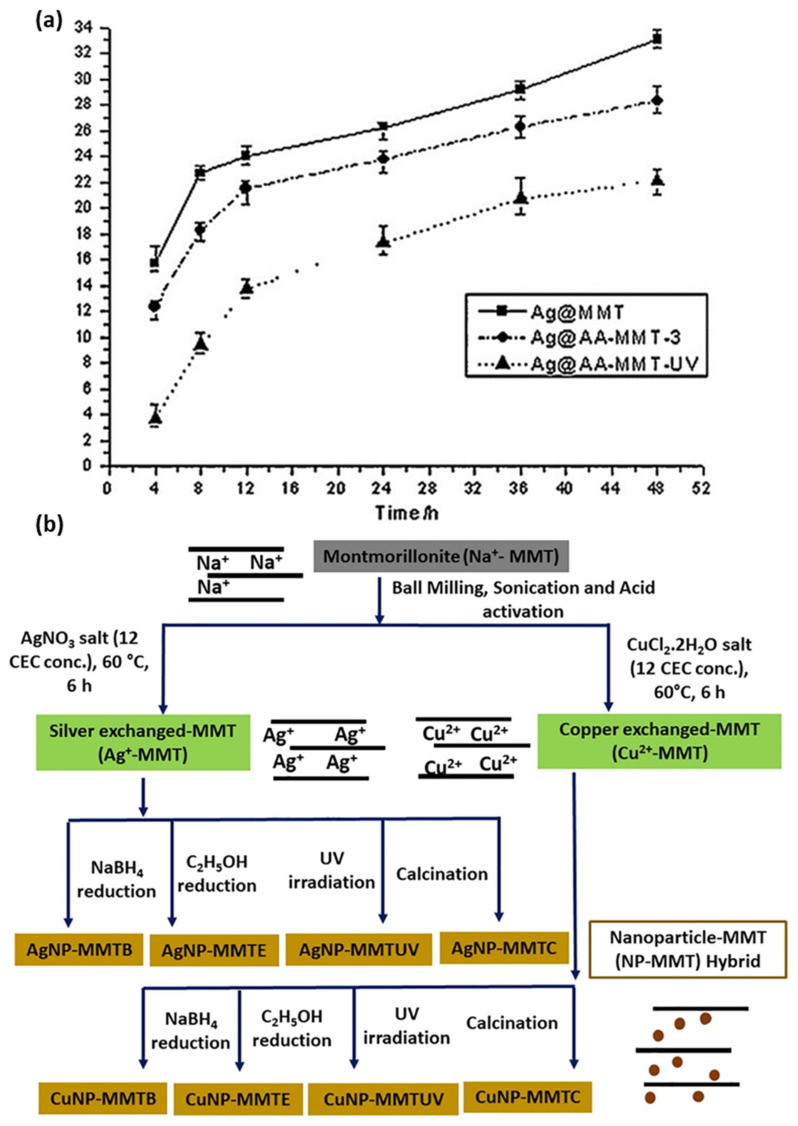
(**a**) Comparison of silver release properties of Ag@MMT, Ag@AA-MMT-3 and Ag@AA-MMT-UV. Adapted from [186] with permission from Elsevier (Copyright 2014). (**b**) Preparation protocol followed for synthesis of Ag/MMT and Cu/MMT nanohybrids. Adapted from [31], with permission from Elsevier (Copyright 2018).

Modification of Ag/MMT by ion exchange method may take a long time to complete the reaction. Therefore, to accelerate the modification reaction of MMT, microwave irradiation has been considered as an alternative method, due to its various benefits such as rapid heating to the reaction temperature, enhanced reaction rates, and formation of novel phases [180]. Taking into account the biological toxicity and the environmental hazard of the residual reducing agent, reduction by different approaches (i.e., UV irradiation [181], and γ-ray irradiation [182], it has been proposed as an alternative way to eliminate the use of toxic reductant and purification steps. Moreover, controlled NP size, with uniform distribution and improved antibacterial activity, is achievable by microwave irradiation method [183,184]. Li et al. proposed that microwave irradiation accelerates the Ag/MMT ion exchange reaction rate, which eliminates the effect of the internal diffusion. As a result, Ag/MMT with a higher silver content and slow release property is obtained. The Ag/MMT shows an inhibition zone 38% larger than that of AgNO_3_, with the same concentration of AgNPs (wt%). In accordance with disk susceptibility test results, MIC values of the Ag/MMT and AgNO_3_ against *S. aureus* ATCC6535 were 1.34 and 1.48 mg/L, respectively; the lower MIC value of the Ag/MMT is due to the fact that the binding of the active agent to the bacteria depends on surface area. The large surface area and the negative surface charge of MMT contribute to the adsorption Ag/MMT on bacteria; consequently, improved antibacterial activity was achieved [185]. The following year, same authors prepared sulfur-containing amino acid (AA) (L-cystine)-modified MMT: Ag@AA-MMT-3, and Ag@AA-MMT-UV, by ion exchange and microwave irradiation method, respectively. Antibacterial activity of Ag@AA-MMT-UV was found to be higher than Ag@AA-MMT-3 and Ag@MMT towards *S. aureus* and *E. coli*, due to slow silver release properties (Figure 3a). Presumably, UV irradiation helps in reducing Ag(I) to Ag(0) [186]. The UV irradiation technique allows silver NP size to decrease while increasing irradiation time [37]. The effect of UV photoreduction in the presence of N-vinyl-2-pyrrolidone (PVP) on silver montmorillonite (Ag/MMT) reduces Ag^+^ into Ag(0), while the slow release property of silver (Ag^+^ and Ag^0^) is possible, particularly for those NPs entrapped in the interlayer of MMT, as NPs desorb more slowly due to the inhibition of MMT sheets. The antibacterial activity of Ag/MMT was attributed to a strong adsorption action of the MMT and electrostatic force between electropositive Ag^+^ and electronegative *E. coli* [187]. Recently, Roy et al. prepared Ag/MMT and Cu/MMT nanocomposites by chemical reduction, calcination, and UV irradiation methods to conduct a comparative study, as shown in Figure 3b. Toxicological properties of Ag/MMT and Cu/MMT hybrid on human RBC and fibroblast cell lines were here reported for the first time [31]. Sun et al. explored the treatment of mixed wound infections, and toxicity experiments based on a quaternary chitosan-coated MMT composite [163]. In 2010, Shameli et al. developed Ag/MMT nanocomposites, with 20–30 nm AgNPs, using γ-irradiation method, without heat treatment and chemical reduction: MMT protected the colloid against aggregation. With increasing γ-irradiation doses, the physical structure of MMT changed to increase holes in the surface of Ag/MMT; the interlayer structure of the MMT suspension gradually solvated e_aq_^−^ electron concentration, which was increased in the solvent while Ag^+^ reduction process, indicating that MMT assisted the γ-reduction process [78]. A drawback could be underlined for the γ-radiation method: a γ source such as ^60^Co is necessary, which might not always be readily available.

**Figure 4:**
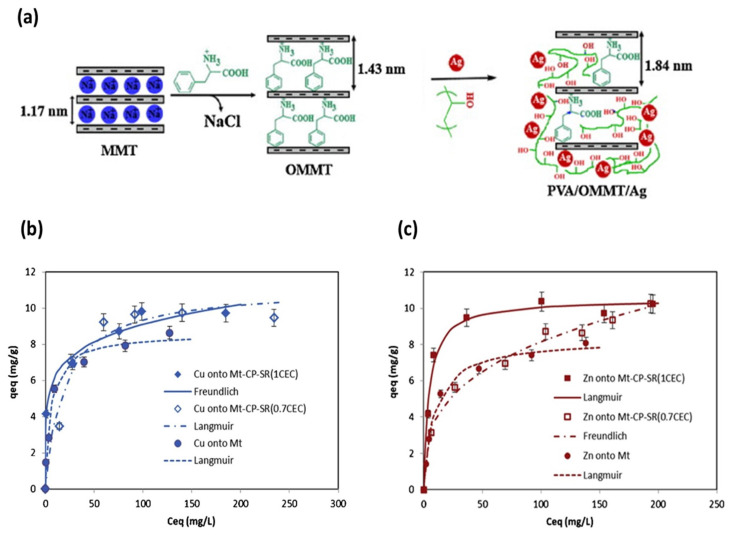
(**a**) Schematic illustrations for preparation of PVA/OMMT/Ag NC films. Adapted from [191], with permission from Elsevier (Copyright 2014). Adsorption of (**b**) Cu^2+^ and (**c**) Zn^2+^ onto the MMT-Na, MMT-CP-SR (0.7CEC), and MMT-CP-SR (1CEC). Adapted from [214], with permission from Elsevier (Copyright 2020, Elsevier).

Shameli et al. applied a UV irradiation reduction method to prepare Ag/MMT/CS bio-nanocomposites (BNCs) without employing reducing agents or heat treatments. It was observed that increasing UV irradiation time from 3 to 96 h led to decreased AgNP size. It is apparent from the inhibition zone test that the Ag/MMT/CS bio-nanocomposites exhibited high antibacterial activity against *S. aureus* and *E. coli*. It is worth mentioning that MMT/CS assisted in the photoreduction process of silver. In addition, it prevented AgNP aggregation [188]. Following a similar approach, Liu et al. explored the synergistic effect of Ag-QCS/OMMT nanocomposite films. Exfoliated MMT adsorbs and fixes microorganisms, while QCS disrupts the cell membrane and allows the AgNPs to react with compounds in the cell wall. The sample showed activity towards Gram-negative bacteria [189]. Analogously, Gabriel et al. reported on the formation of small-sized and homogeneously distributed AgNPs on modified CS/MMT films, with outstanding antibacterial performance against *E. coli* and *B. subtilis*. The synergistic effect of several types of modified chitosan such as Ch30d (viscosity-average molar mass of 30,000 g mol^−1^; 98% deacetylation degree), Ch30-DEAE (diethylaminoethyl), Ch30-DEAE-Dod (dodecyl), and clay concentration on nanocomposite films has been discussed. On the one hand, DEAE groups in polymer chains increase chelation sites, which facilitate Ag^+^ reduction after the UV irradiation process. On the other hand, Ch30-DEAE-Dod is an amphiphilic compound which can act as a surfactant, controlling Ag NP size and distribution [190]. In another study, Mallakpour et al. prepared poly(vinyl alcohol)/organoclay/silver (PVA/OMMT/Ag) nanocomposite films with different silver compositions by ion exchange method under ultrasonic irradiation (Figure 4a); AgNPs were uniformly dispersed in the polymer matrix, which improved the thermal stability of the proposed sample [191]. Nonetheless, to the best of our knowledge, no significant effort has been devoted to the development of Cu/MMT nanocomposites by irradiation method.

### 2.8. Structural Investigation, Acid Treatment, and Adsorption Study Based on Ag/MMT Nanocomposite

Molecular modelling has been used to explore the structure of Ag/MMT [192]; structural study of Ag/MMT being fundamental to determine the exact localization of silver [193]; acid treatment eliminates the use of reducing agent [9,25]. The investigation of metal and metal oxide NPs adsorption on substrates could play a significant role in determining the antimicrobial performance and mechanism. For example, Zarei and Barghak et al. performed adsorption capacity tests and found that Na/MMT could remove a significant amount of AgNPs (more than 70 mg/g) in 10 min at pH 7. Further, it was proven that the Langmuir model is more accurate than the Freundlich one, describing the adsorption of AgNPs on MMT [194]. In this scenario, Syafiuddin et al. discussed the adsorption of AgNPs onto several types of adsorbents, such as core-shell mesoporous silica, glass beads, iron oxide magnetic particles, poly(ethylenimine) functionalized core-shell magnetic microspheres, and Fe_3_O_4_/polydopamine core-shell ones [195]. Interestingly, Solarte et al. studied hexadecyltrimethylammonium (CTA^+^) and Ag adsorption on MMT, with a synergistic effect towards *S. aureus*. However, the authors underlined that the synergistic effect is not visible for *E. coli* [196].

In 1995, Keller-Besrest et al. first investigated the structure of Ag/MMT composite by extended X-ray absorption fine structure (EXAFS) spectroscopy, and explored triangular silver cluster structures. The study apparently showed MMT containing Ag ions, AgNPs, and Ag_2_O with evidence of aggregation of the silver atoms in triangular clusters [193]. Previously, Busolo et al. described ionic Ag-exchanged MMT without the presence of AgNPs [197]. Later, Clegg et al. emphasized the production of AgNPs on bentonite clay surfaces [198]. With the aim of developing smaller AgNPs, Shabanzadeh et al. designed an artificial neural network (ANN), and outlined that AgNO_3_ concentration plays a significant role in determining NP size [199]. In another interesting study, Tokarsky et al. exploited molecular modelling (force field) for the investigation of the structure, stability, and adhesion energy of silver NPs on MMT clay. Further, they correlated the modelling data with results of spectroscopic analysis. It was found that AgNP adhesion on the substrate becomes weaker while increasing NP size and thickness. Van der Waals coulombic forces and bond length were taken into account into the model [192]. Following that, Valášková et al. used molecular modelling to investigate interlayer space arrangement of vermiculite during the interaction with α-Fe_2_O_3_ [200].

Tian et al. incorporated Ag/MMT into polycarbonate (PC); after that, the prepared samples were treated with 1,4-dimethylbenzene in order to obtain functional Ag/MMT/PC composite with super hydrophobicity. The combination of inorganic materials and polymers leads to excellent bio- and catalytic activity on the degradation reaction of methylene blue [201]. Roy et al. developed Ag/MMT by cation exchange method, and subsequent acid treatment, obtaining spherical AgNPs on MMT. Acid-activated Ag/MMT exhibited higher antibacterial efficacy than the untreated one, but lower than AgNPs, towards *E. coli* and *S. aureus*. Acid-treated Ag/MMT also showed a higher uptake of silver ions, which led to improved cation exchange capacity. It was hypothesized that the acid treatment leads to structural alterations such as an enrichment in crosslinked mesopores. Under these conditions, a higher negative charge was observed [25], which correlates with a higher surface area and pore volume [202,203]. In order to avoid NP agglomeration and to achieve the controlled slow release of bioactive ions, Ag/MMT was embedded in glass matrix [204]. It is worth noting that the study further led to the exploration of the potential application of inorganic metal nanomaterials in therapeutic and tissue engineering fields [205,206].

### 2.9. Adsorption Properties of Cu/MMT

Ma et al. showed the effect of temperature and ionic strength on the adsorption of methylene blue (MB) onto MMT- and Cu^2+^-exchanged MMT (CEM) [207]. It has been reported that Cu/MMT effectively reduces aflatoxin toxicity [208,209]. Moreover, Cu/MMT nanocomposite increases the adsorption of *Aspergillus* fungi aflatoxin B1 (AFB1), which is presumably due to the formation of chelation complexes of both Cu^2+^ and Ca^2+^ ions with the β-di-carbonyl system of AFB1 [209]. In 2009, Özdemir et al. reported the desorption of Cu^2+^ from the MMT surface in acidic solution at pH 2.0. The study suggested that the antibacterial activity of the cation mainly arose from adsorbed copper ions. It should not be left unmentioned that the low cation desorption in the aqueous medium caused a less negative impact on the environment [210]. Alternatively, Guo et al. explained the adsorption properties Cu/MMT composite towards *E. coli* K88. The rate of *E. coli* K88 adsorption on Cu/MMT composite was found to be higher in an acidic medium than in an alkaline one [211]. Based on this, Gu et al. developed algae removal nanocomposite with 5 to 10 nm sized Cu_2_O, by the reduction of MMT-absorbed Cu^2+^ using glucose and ethylene glycol as reductants [212]. Differently, Yan et al. synthesized MMT-reduced graphene oxide–Cu NPs (MMT-rGOx-CuNPs) and postulated that the nanocomposite adsorbed *S. aureus* more strongly than *E. coli*. Furthermore, based on bacteria morphological changes, they claimed that the outer membrane (OM) of *E. coli* defended cells from external attack compared to *S. aureus*. On the course of action, MMT-rGOx was selected as carrier; however, the role of rGOx in the nanocomposite and its effect on bacteria was not clear [213]. Recently, Özdemir et al. reported that the addition of N-lauroylsarcosinate (SR) to cetylpyridinium (CP) intercalated montmorillonite (MMT-CP) increased the adsorbed amount of Cu^2+^/Zn^2+^. The adsorbed amount of Cu^2+^ was found slightly higher than that of Zn^2+^, as shown in Figure 4b,c. The reason could be attributed to the ionic properties of Cu^2+^ and hydration degree, which ascertains the binding forces between the Cu^2+^/Zn^2+^ and MMT surface. The Langmuir isotherm model refers to homogeneous adsorption (all sites possess equal affinity for the adsorbate). The Freundlich model, instead, could be applied from a monolayer to an extended multilayer adsorption, with non-uniform distribution of affinities over the heterogeneous surface. Furthermore, the authors evaluated the effect of Cu^2+^/Zn^2+^ immobilization on OMMT, in which the structure was formed with cationic and anionic surfactants [214].

### 2.10. Adsorption Properties of Zn/MMT

Dakovic et al. determined the AFB1 adsorption isotherm by Zn/MMT at pH 3, with a capacity, which was higher than pristine MMT one by a factor of about 50%. This is probably due to the interactions between AFB1 and Zn ions in both the interlayer and at the surface [215]. Recently, Egirani et al. reported ZnO coating on Acros Organics (ACOR) MMT as an effective adsorbent for lead ion (Pb^2+^) removal. The study suggested that protonation was attenuated by the ZnO coating on ACOR MMT. The presence of the ZnO coating covered the acidic sites on the edges and planar surfaces of ACOR MMT. Three steps of mass transfer including film diffusion, adsorbent surface diffusion, and intraparticle diffusion were observed [216]. Previously, Ma et al. explored the adsorption properties of Cu^2+^–ZnO/cetylpyridinium (CP)-MMT in vitro against pathogenic *E. coli*. Further, they postulated that the bacterial adsorption on the (CP^+^)-MMT surface led to increased permeability, prolonged hydrophobicity and the reversal of surface charge from negative to positive, with consequent higher percentages of *E. coli* adsorption [217].

### 2.11. Ag/MMT, Cu/MMT, and ZnO/MMT Nanocomposite by Electrochemical, Plasma resonance, and Radio Frequency (RF) Methods

**Figure 5:**
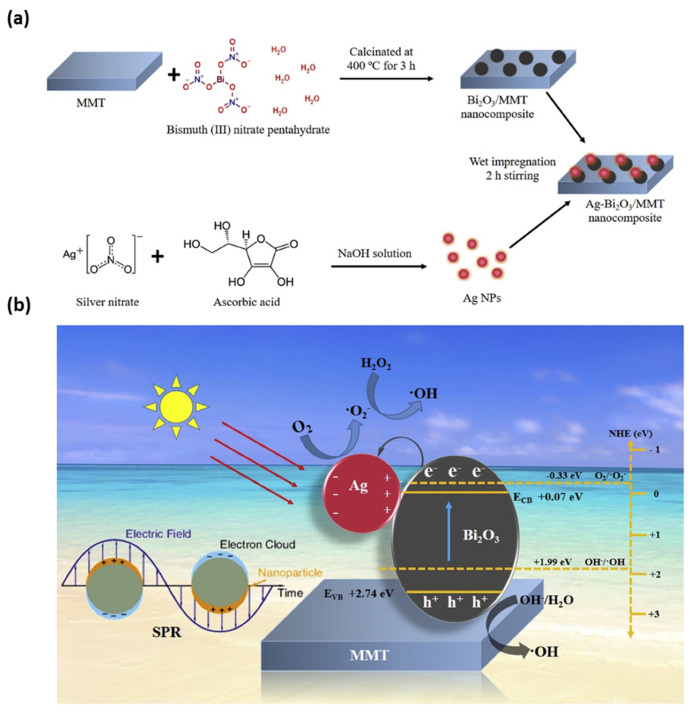
(**a**) Schematic diagram of the synthesis process of Ag–Bi_2_O_3_/MMT nanocomposite and (**b**) schematic representation of electron–hole separation of Ag–Bi_2_O_3_/MMT composite under visible light irradiation. Adapted from [221], with permission from Elsevier (Copyright 2020, Elsevier).

Taking environmental and health issues into account [218], the development of novel synthetic routes for the fabrication of multicomponent coatings with uniform thickness, strong adhesion to the substrate [219] with improved antimicrobial performance, is highly needed. In this regard, electrochemical synthetic routes are considered well advanced, inexpensive, and eco-friendly, and allow the obtaining of NPs with fine morphological and dimensional control and with high purity [72]. Huang et al. fabricated AgNPs by facile electrochemical method in the presence of Na-MMT; antibacterial activity of AgNP/MMT was investigated against *S. aureus* ATCC 6538P. In particular, AgNPs were prepared by a combination of two schemes, i.e., the electrochemical synthesis of stabilized metal clusters, and simultaneous reduction in AgNPs using methanol as the reducing agent. MMT served as both stabilizer and support during the electrochemical process. At reduction potentials of 5, 10, 15, and 20 V, the formed AgNPs in the aqueous solution had average sizes of 4, 6, 15, and 20 nm, respectively. In the prepared AgNP/MMT in aqueous solutions, AgNPs on the MMT surface had slightly higher average sizes of 5, 8, 17, and 25 nm [218]. In 2019, Iconaru et al. applied a radio frequency (RF) magnetron sputtering method to deposit MMT and Ag/MMT layers on Si substrates, studying their antifungal activity towards *Candida albicans* ATCC 101231. Both MMT and Ag/MMT layers showed good activity: after 72 h, the *Candida albicans* ATCC 101231 growth was inhibited significantly due to the presence of silver ions and their controlled release [220]. Tun et al. applied thermal and wet impregnation methods to fabricate functionalized plasmonic Ag-Bi_2_O_3_/MMT nanocomposites (Figure 5a), whereby surface plasmon resonance (SPR) of AgNPs appeared, and can be seen in Figure 5b [221].

## 3. Comparison of Antimicrobial Performance and Mechanism among Ag/MMT, Cu/MMT, and Zn/MMT Nanocomposite

**Figure 6:**
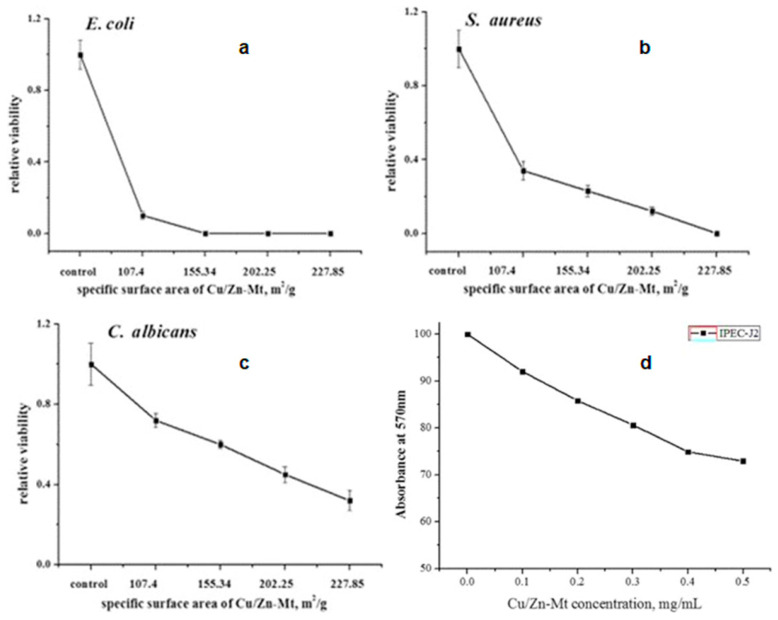
(**a**–**c**) The antimicrobial activity of Cu/Zn-MMT-2 with different specific surface area, (**d**) the cytotoxicity of Cu/Zn-MMT. Adapted from [229], BMC part of Springer Nature, distributed under the terms and conditions of the Creative Commons Attribution (CC BY) license.

Özdemir et al. showed that Cu^2+^, Ag^+^-exchanged, and Ag^0^-covered MMT exhibited excellent antibacterial performance against *P. aeruginosa* and *S. aureus*. On the contrary, Zn^2+^/MMT was powerful only against *S. aureus*, which suggested the potential use of Zn/MMT instead of Ag/MMT for applications towards *S. aureus* [46]. In another interesting study, the antibacterial (*E. coli*) and antifungal (*Pycnoporus cinnabarinus* and *Pleurotus ostreatus*) activities of Ag/MMT, Cu/MMT, and Zn/MMT were evaluated. The study showed the strongest antibacterial performance for Ag/MMT and Ag^+^ ions. Cu/MMT was as effective as free Cu ions. Zn/MMT showed the weakest performance, similar to that of Zinc ions. Zn is an essential element, and can enhance bacterial growth at low concentrations. The antifungal effect of Zn/MMT and Cu/MMT was found to be higher than the inhibition by silver ions and Ag/MMT [222]. Previously, Kim et al. investigated the antibacterial effects of silver/hydroxyapatite (Ag-HA), Cu-HA, and Zn-HA composites. The antimicrobial ceramics (AC) based on HA were made by a wet chemical process. The authors noted an antimicrobial effect against *E. coli* in the case of Ag^+^/AC composite, whereas no antimicrobial effect was observed for Cu/AC and Zn/AC. The reason behind this inconsistency was not vivid [223]. The use of MMT-based adsorbents is considered as a potential technique to remove bacteria from contaminated water. In this regard, Al^3+^, Ag^+^, Cu^2+^, and Zn^2+^ played a significant role in antibacterial processes when the clay mineral was brought into contact with bacteria. The study suggested that the antibacterial effect of the metal cations is localized on the clay surface [224]. Recently, Roy et al. reported antimicrobial activity for metals containing HDPE-MMT composites, towards *E. coli* and *S. aureus*. In this regard, the efficiency was classified as follows: HDPE/Ag/MMT>HDPE/Cu-MMT>HDPE/Zn-MMT [225]. Alarmingly, the complex structure of bacteria continually adapts to the mechanism of action of well-known single metal and metal oxide NPs. Incorporation of dual active phases in the MMT matrix could be a promising way to find an effective alternative to combat colonies of drug resistant bacteria. Due to different modes of antimicrobial action of individual material in synergistic composites, they can attack peculiar bacteria at different stages of their life cycle. In this regard, researchers are focusing their attention on preparing multicomponent nanocomposites for antimicrobial application [226]. For example, Ma et al. impregnated Cu^2+^ and ZnO on MMT and further modified it with cetylpyridinium ions to investigate the antibacterial mechanism towards pathogenic *E. coli* and *Salmonella typhimurium*. The prepared Cu^2+^–ZnO/cetylpyridinium–montmorillonite (CZCM) nanocomposite attenuated the bacterial wall thanks to the interactions between proteins and enzymes of the cell wall. Moreover, the composite influenced the efflux of intracellular nutrients such as K^+^ and enzymes. The formed CZCM nanocomposite negatively affected the tricarboxylic acid pathway of the bacterial respiratory metabolism [227]. Following that, the same authors embedded Cu^2+^ and ZnO into zeolite in order to investigate its antibacterial performance against *E. coli* and *S. aureus* [228]. In 2017, Jiao et al. showed that Cu-Zn/MMT exhibited improved antibacterial and antifungal performance than bare Zn/MMT and Cu/MMT. The reason behind that could be due to the synergistic effect between Cu and Zn. Moreover, the antimicrobial activity was observed due to the enhancement of the specific surface area, as shown in Figure 6a–c [229]. In a recent study, carboxymethyl cellulose (CMC)-based films incorporating MMT modified with Ag and Cu ions were found to be less effective than Ag- or Cu-modified CMC-Cloisite 30B (additive for plastics) films against *S. aureus* and *E. coli*, and had a lower tensile strength, which is probably due to the better compatibility of C30B clay NPs in the biopolymer matrix than bare MMT. Particularly, Gram-positive bacteria were found to be more sensitive to these NPs than Gram-negative bacteria [230].

## 4. Selected Cases of Study about Antimicrobial Applications of MMT-Based Materials

It is well known that Ag, Cu, and ZnO NPs show effective antibacterial activity [19,20,23]. The load of metal NPs on the surface of MMT and its incorporation within polymeric matrices (agar hydrogel, PVA, glassy matrix, calcium alginate, CA, CMC, konjac glucomannan/polycaprolactone, agar–CMC, (QCS)/(OMMT), CS/pPDA, polydimethylsiloxane (PDMS), KGM, PCL, poly (butylene adipate-co-terephthalate) (PBAT)) causes higher water uptake, improved thermal stability, and mechanical properties, along with changes in barrier and permeability properties. Most importantly, those nanocomposites exhibited improved antimicrobial activity towards a broad variety of pathogens, including *E. coli*, *S. aureus*, *M. luteus*, and *I. orientails*. Therefore, those nanocomposites are considered as potential active agents in food packaging, food bacteriology, agriculture, food technology, food preservation, biomedical field, wastewater treatment, clothing, marine biofouling on ship (Table 1) [38,39,40,97,126,128,144,149,171,173,204,231,232,233,234,235,236]. Pristine Ag/MMT is efficient in food technology since it is capable of reducing microbial viability [127]. Zhang et al. developed Ag/OMMT by a one-step solution–intercalation technique, and postulated that the composite is able to kill 100% of *S. aureus*, *E. coli*, and *C. albicans*, within 2 h, in solution; the approach is convenient to prepare surface-modified nanocomposites for adhesives, plastics, and paints manufacturing industries [136]. Functionalized plasmonic Ag-Bi_2_O_3_/MMT nanocomposites are potent in removing antibiotic pollutants and dyes from wastewater [221]. In another study, the slow release of silver chelators from (Ag^+^(6-BAP)_2_) MMT composite is attributed due to the electrostatic adsorption state in the deepest part of the nanolayers, making MMT clay a suitable host material for the delivery of bactericidal compounds [76]. Ag/MMT suspension can also be useful to modify bacterial cellulose (BC) membranes: MMT or Ag/MMT can diffuse through the BC matrix and adsorb on the surface of the polymeric networks. Ag/MMT/BC showed good properties towards wound healing, inhibiting biofilms of *S. aureus* and *P. aeruginosa* [237]. In 2016, Cu/MMT nanocomposite exhibited antibacterial performance towards *S. aureus* and *E. coli*, with excellent stability in water, which allowed for the consideration of the sample as a potential agent for wastewater treatment [152]. Later, Cu-Zn/MMT was found to be efficient in animal husbandry application because of its outstanding antimicrobial activity and relatively low toxicity [229]. The preparation of Cu-Zn/MMT hybrid nanocomposites by developing or adapting green, facile, scalable, and cost effective methods could open new perspectives for their real life application in the fight against antimicrobial resistance or biofilm. In a recent review, antimicrobial activity of nanomaterials towards foodborne pathogens in polymeric food packaging has been highlighted [238].

## 5. Environmental and Toxicity Issue

Most environmental and toxicity studies focus on Ag, Cu, and ZnO colloids, and a very few studies report on Ag/MMT, Cu/MMT, and Zn/MMT nanocomposites. The nanotoxicology of Ag-, Cu-, and ZnO-containing nanomaterials has been the subject of several review papers [19,242,243,244,245,246,247,248,249]. In this present review, we have focused on supported nanophases, on substrates such as MMT, implementing, embedding, and impregnating Ag ions, AgNPs, Cu ions, CuNPs, and ZnONPs. Nanoparticles and microparticles supporting smaller nanophases exhibit lower toxicity, as to the bigger size of the overall size of the nano-/micro-structure. The cytotoxic effect of metal NPs on different mammalian cell lines has been widely investigated [250,251,252]. Nonetheless, the impregnation of NPs on MMT which is a non-toxic material and has a synergistic effect on its cytotoxic behavior, leads to the synthesis of novel cyto-compatible antimicrobial nanocomposites [31,204,220]. It is well known that NP toxicity depends on the particle properties such as size, shape, charge, surface energy, and chemical composition, and on the type of involved organisms. Antimicrobial NPs might cause adverse toxic effects in human organs and could damage DNA [253]. It is also proven that NPs smaller than 30 nm can easily penetrate skin, reaching the deepest layers of the stratum corneum and the outermost surface of the epidermis, and nasal/pharyngeal mucosa [71,254]. Due to uncontrolled release behavior, antimicrobial NPs could be toxic. When working with antimicrobials providing a controlled release, the metal ion concentration should be adjusted below 0.1 mg/L, which can be tolerated without health risks [51,76,255]. However, this concentration can be changed depending on the ways of interaction (skin penetration, respiratory tract, ingestion), type of metal, and prolonged or short-term exposure.

Toxicological properties of Ag/MMT and Cu/MMT hybrids on human RBC and fibroblast cell lines have been reported. Whereby, at a 10 μg/mL concentration, Ag/MMT exhibited higher hemolysis (55.2 ± 2.2%) than Ag/MMTUV (38.3 ± 1.5%), because of a smaller NP diameter and higher loading. In contrast, at 400 μg/mL concentration, Cu/MMTB and Cu/MMT-UV exhibited 30.1 ± 1.2% and 15.0 ± 0.6% hemolysis, respectively. In relation to the same conditions, the cytocompatibility of Ag/MMTUV and Cu-MMUV was found to be 96.6 ± 3.1% and 100 ± 12%, respectively. Hence, it appears that Cu/MMT hybrids are safer than Ag-based nanocomposites within human cell lines [31]. Ag^+^/MMT/sutures, prepared by a hybrid method, exhibited a hemolytic activity of 4.8% on blood cells and did not show significant cytotoxicity to human endothelial cell meridians, even after 7 days of continuous incubation [125]. Cytotoxicity studies on MMT and Ag/MMT showed that they did not reveal any considerable toxicity towards HeLa cells, in which viability decreased from 96% at 24 h, to 92% after 72 h of incubation [220]. In addition, Lal et al. investigated that MMT–insulin–PLGA composites were non-toxic towards human embryonic kidney HEK-293 [256]. Polycaprolactone)/MMT (PCL/MMT) composites do not damage plasma membrane. It was noted that the presence of MMT did not significantly affect the viability of fibroblasts [257]. Hemolytic activity of Ag/MMT/Lys towards human erythrocytes and the effect of aluminum oxide content on MMT was investigated. Increasing Al_2_O_3_ content in MMT caused the increase in negative surface zeta potential, resulting in the repulsion of Ag/Lyz NPs from the MMT surface. Consequently, silver content decreased at the MMT surface. This phenomena led to an increase in hemolytic activity (e.g., cytotoxicity) of the sample, which eventually reduced its antibacterial activity [146]. Previously, Ag/MMT nanomaterials were embedded in glassy matrix, which avoided NP agglomeration and mitigated toxicity in human cells, by slowing the release of nanomaterials [204]. HDPE-Zn/MMT nanocomposites exhibited 100% cytocompatibility in MTT assay, which suggests their inertness to dermal fibroblast cells, whereas HDPE/ZnO nanocomposites exhibited mild hemotoxic and cytotoxic behavior, since modified MMT/polymer matrix, intercalation, and exfoliation, affect the nanocomposite morphologies [175]. The in vitro and in vivo analyses of polymeric nanocomposite (Polyethylene/Ag/MMT) showed RBC hemolysis protection and cytocompatibility, and no viable cell count reduction to human erythrocytes and to dermal fibroblast cell lines [134]. It has been found that increasing the concentration of Cu-Zn-MMT causes moderate cytotoxicity to intestinal porcine epithelial cell line (IPEC-J2), as can be seen in Figure 6d [229]. In another study, the same authors showed that supplementing Cu-Zn-MMT in the diet of animals increased (*p* < 0.05) the Cu and Zn concentrations in serum, jejunum, and ileum mucosa. In the meantime, this enhanced the height of villus, while decreasing the depth of crypt [258]. Zhang et al. found the same trend using the zinc-bearing palygorskite diet supplement [259]. The study of toxicity and immunological activity of AgNPs in Swiss mice by using zerovalent Ag load on MMT showed no significant changes in mice behavior during 96 h of observation, which corresponded to no neurotoxic effects [260]. Cytotoxicity of the Nacre-like konjac glucomannan–Montmorillonite (KGM–MMM) nanocomposites was evaluated using RAW264.7 cells (monocyte/macrophage-like cells) [97]. Polymeric anthocyanin-containing OMMT has been found to be an efficient pH-sensitive antioxidant for nano-packaging systems. However, the toxicity of polymeric anthocyanin/OMMT nanocomposites actually limit their application in the food packaging system [261]. In contrast, due to beneficial interactions of AgNPs with living structures and their non-toxic effects on healthy human cells, they are considered a potential agent for various biomedical products [262]. The incorporation of non-toxic NPs into MMT open a new perspective.

## 6. Perspectives

Montmorillonite-based nanoantimicrobials open new perspectives in the manufacturing of medical devices, the textile industry, wastewater treatment, and food packaging. Either the in situ or ex situ preparation of metal and metal ions into and onto the MMT matrix, and the inclusion of bioactive polymers have been explored to control the size and shape of NPs, control the release of bioactive specimens, prevent NP agglomeration, develop synergistic and improved nanoantimicrobials, and address toxicity problems. Several approaches have been proposed: ion exchange, chemical reduction (involving surfactants, caping agents, chemical salts, solvents, plant extracts, biological species, γ-rays, microwave and UV irradiation), hydrothermal synthesis, solution casting, RAFT polymerization, electrospinning, dipping and rolling, thermal treatment, the dissolution–intercalation method, calcination and grinding, polycondensation, low-temperature solvothermal synthesis, melt compounding, electrochemical synthesis, plasma resonance, and radiofrequency.

Most of the published articles outline that nanocomposite antimicrobial performances depend on the slow release of active agents/ions, ion binding capabilities to bacterial functional groups, size, shape, the uniform distribution of NPs into MMT, increased electrostatic interaction between nanocomposite and bacteria, and a synergistic effect by using multicomponent materials. However, the role and mechanism of action, for each single component within the composites, are worth further investigation. Notably, the comparison of real performances among reported nanocomposites against bacteria is not accessible, due to the use of different growth conditions, bacterial concentrations, and the MMT/NP ratio. A small environmental change could cause unusual effects on bacteria, leading to ambiguous antimicrobial test results. In most of the papers, detailed spectroscopic study is missing: the latter is particularly important to understand the long time stability of nanocomposites.

In general, it is apparent that MMT-based NAMs are highly active towards Gram-positive and Gram-negative bacteria and can significantly inhibit and eradicate pathogens and harmful biofilms. Antimicrobial colloidal NPs have attracted enormous attention, and MMT-based materials sound mature for a wider use in the mentioned application fields. They could be dispersed in almost all kinds of organic and inorganic solvents, paving the way to the preparation of a novel class of hybrid materials to be used as additives in food and cosmetic industries, hard coatings, and biomedical devices.

## 7. Conclusions

This review provides a selection of the most convenient and easily scalable nanoantimicrobial fabrication routes, with a peculiar attention on the effect of synthetic parameters on the efficacy of these MMT-based composites against different bacterial strains, as a function of the final applications. After this literature overview, we can conclude that MMT is generally considered to be cost effective and easily available, and possesses a large surface area and highly negative surface charge, which effectively promotes electrostatic interaction between nanocomposites and bacteria. NP-modified MMT can be easily prepared on polymer matrix by either common polymer fabrication techniques or hybrid methods. NP-containing MMT can be effectively applied as coating on plastic, metal, food packaging, medical device, and textiles. Prolonged use of NPs/MMT/polymer nanocomposites with reduced toxicity is possible. Altering the surface charge of nanocomposites can promote electrostatic interactions with bacteria, improving the material antimicrobial activity. MMT could be considered a unique support which can accommodate multiple components and functionalities; hence, it could support the design of new materials and technological devices for industrial applications.

## Figures and Tables

**Table 1:** Summary of literature of nanocomposite prepared by several methods for potential application in different sectors.

Type of Nanocomposite	Methods of Preparation	Application	Antimicrobial Result/Improved Properties	References
Ag/OMMT/QCS-QOMA	One-step approach.	Medical device and household.	Improved mechanical properties and confirmed activity towards *E. coli*, *S. aureus*, and fungi.	[39]
(Ag^+^(6-BAP)_2_)/MMT	Ion exchange method and carbothermal reduction.	Delivery of bacteriocidal compounds.	Slow release of silver chelate is confirmed.	[76]
(Ag-Nacre-like KGM)/MMT	Vaccum filtration and in situ reduction method.	Biomedical field.	Good mechanical properties and improved light transmission.	[97]
Ag/MMT/PCL	Reduction method.	Wastewater treatment.	Confirmed antibacterial activity towards *S. aureus* and *E. coli*	[103]
Ag/MMT/glassy matrix	Embedded.	Agriculture and food technology.	Confirmed activity towards *E. coli*, *M. luteus*, and *I. orientails*	[204]
Ag/MMT/PVA	Electrospinning method.	Food bacteriology.	Effective against *S. aureus* (ATCC6538) and *E. coli* (ATCC25922), improvement in thermal stability.	[149]
Ag/MMT/agar hydrogel	Ion exchange and thermal treatment method.	Food packaging.	Activite against selected spoilage microorganisms.	[126]
Ag/MMT	Ion exchange and thermal treatment method.	Fresh fruit salad.	Shelf life increase in fruit salad and reduced microbial viable count.	[127]
Ag/OMMT	One-step solution-intercalation technique.	Adhesives, plastics, and paints manufacturing industries.	Confirmed that in 2 h, 0.0125 mg/mL Ag/OMMT could kill 100% of *S. aureus*, *E. coli*, and *C. albicans* in solution.	[136]
Ag/MMT/Agar–CMC	Solution casting method.	Food preservation.	Improved mechanical and antimicrobial properties.	[144]
Ag/MMT	Aqueous extract of Acetabularia acetabulum.	Marine biofouling on ship.	Active against *S. aureus* and *E coli*	[236]
Ag-Bi_2_O_3_/MMT	Thermal and wet impregnation methods.	Refine antibiotic pollutants and dye from wastewater.	Showed outstanding visible light induced photocatalytic activities for tetracycline (TC) antibiotic and RhB degradation.	[221]
Ag/MMT/BC	Ion exchange, solution casting.	Scaffolds for wound dressing	Active against biofilms of *S. aureus* and *P. aeruginosa.*	[237]
Calcium–alginate coating loaded Ag/MMT	Ion exchange method.	Fresh-cut carrots	Improvement of sensory attributes, spoilage microorganisms reduced, Extension in shelf life of carrots.	[128]
Cu^2+^/MMT/CA	Solution casting.	Food packaging	The change in the oxygen barrier properties, confirmed antibacterial performance against *E. coli*.	[38]
Cu^2+^/MMTCarbon Paste Electrode (CPE)	Ion exchange and electrochemical method.	Detect propineb pesticide in river and sea water.	Confirmed sensitivity towards pesticide.	[239]
Cu/MMT	Alkaline ion exchange.	Wastewater treatment.	Confirmed antimicrobial activity against *S. aureus* and *E. coli*, with excellent stability in water.	[152]
Cu-Zn/MMT	Ion exchange reaction.	Animal husbandry.	Higher antimicrobial activity towards *E. coli*, *S. aureus* bacteria; and Candida albicans fungi, relatively low toxic.	[229]
Cu-Alpill/MMT	High power ultrasonic treatment and calcining.	Wastewater treatment.		[240]
Cu^2+^/MMT/CMC	Ion exchange and spray coating.		Active against Erwinia carotovora in Potato (*Solanum tuberosum* L.)	[164]
ZnO/MMT/CMC	Solution casting.	Food packaging.	Improved functional characteristics, and enhanced resistance to water vapour permeability, glass transition temperature increased, higher antibacterial activity against *S. aureus* than *E. coli*.	[171]
PCL/halloysite (HNT)/MMT	Heat treatment, precipitation	Beef meat packaging.		[241]

## Data Availability

Data is contained within the article.
